# Effects of Honey and Its Mechanisms of Action on the Development and Progression of Cancer

**DOI:** 10.3390/molecules19022497

**Published:** 2014-02-21

**Authors:** Omotayo O. Erejuwa, Siti A. Sulaiman, Mohd S. Ab Wahab

**Affiliations:** Department of Pharmacology, School of Medical Sciences, Universiti Sains Malaysia, 16150 Kubang Kerian, Kelantan, Malaysia; E-Mails: sbsamrah@kb.usm.my (S.A.S.); msuhaimi@kb.usm.my (M.S.A.W.)

**Keywords:** cancer, honey, anticancer, antimetastatic, antiproliferative, antitumor

## Abstract

Honey is a natural product known for its varied biological or pharmacological activities—ranging from anti-inflammatory, antioxidant, antibacterial, antihypertensive to hypoglycemic effects. This review article focuses on the role of honey in modulating the development and progression of tumors or cancers. It reviews available evidence (some of which is very recent) with regards to the antimetastatic, antiproliferative and anticancer effects of honey in various forms of cancer. These effects of honey have been thoroughly investigated in certain cancers such as breast, liver and colorectal cancer cell lines. In contrast, limited but promising data are available for other forms of cancers including prostate, bladder, endometrial, kidney, skin, cervical, oral and bone cancer cells. The article also underscores the various possible mechanisms by which honey may inhibit growth and proliferation of tumors or cancers. These include regulation of cell cycle, activation of mitochondrial pathway, induction of mitochondrial outer membrane permeabilization, induction of apoptosis, modulation of oxidative stress, amelioration of inflammation, modulation of insulin signaling and inhibition of angiogenesis. Honey is highly cytotoxic against tumor or cancer cells while it is non-cytotoxic to normal cells. The data indicate that honey can inhibit carcinogenesis by modulating the molecular processes of initiation, promotion, and progression stages. Thus, it may serve as a potential and promising anticancer agent which warrants further experimental and clinical studies.

## 1. Introduction

Cancer refers to an unrestrained growth of cells which may exhibit malignant behavior. It is one of the leading causes of death worldwide [1]. The most recent global incidence of cancer was estimated at 28.8 million in 2008 [2]. This rising prevalence is driven by certain factors, such as the adoption of a Western lifestyle in several developing countries, ageing populations, increased awareness and improved screenings and diagnosis of cancers [3]. Out of the 7.6 million cancer deaths recorded in 2008, 2.9 and 4.7 million deaths occurred in economically developed and economically developing countries, respectively [1]. The impact of this disease is more visible in developing countries. Several environmental factors such as smoking, physical inactivity, infections and diseases including obesity and diabetes are key risk factors for cancer [4]. The process of cancer development comprises three key stages: initiation, promotion, and progression. Initiation, which is the first stage of carcinogenesis, involves irreversible genetic damage and is characterized by accumulation of mutated DNA [5]. This is followed by promotion stage which is the proliferation of mutated cells. It is characterized by excessive growth of mutated cells and additional genomic alterations of the replicated cells giving rise to a benign mass of abnormal cells known as a tumor [6]. Then the progression stage which entails metastasis of cancer cells to distant sites (tissues and organs) through the lymphatic or circulatory systems [5,6]. Besides the limitations of current cancer management (surgery, chemotherapy and radiotherapy), available cytotoxic drugs are not easily affordable and available in some places (especially in developing countries), and their use is also associated with a number of undesirable side and adverse effects [7,8]. As a consequence, a large proportion of the population prefers to patronize complementary and alternative medicine (CAM). Even though CAM has its own limitations, it does however have a number of advantages (such as affordability, availability and lower side effects) compared to the synthetic or standard drugs [9]. The increased public interest in the utilization of CAM can be linked to the increased research data which attribute some health benefits to CAM.

Generally, CAM refers to diverse treatment approaches (medical products and practices) that are not an integral part of standard medicine and may include dietary components, supplements, herbal preparations, naturally-derived products and even lifestyle changes [10]. There has been an increased awareness and use of CAM among cancer patients [11]. One of such products that have become an important component of CAM is honey, a natural substance formed from nectar by honeybees. Honey exerts several biological activities such as antibacterial, hepatoprotective, anti-inflammatory, hypoglycemic, antioxidant and antihypertensive effects [12,13,14,15,16,17,18]. Besides its sugar content (monosaccharides, disaccharides and oligosaccharides), honey consists of several biologically active constituents such as flavonoids and phenolic compounds, vitamins, trace elements, amino acids and proteins as well as certain enzymes including glucose oxidase, invertase and catalase [19,20]. Even though the use of honey dates back to ancient times, the last decade has witnessed an astronomical increase in the amount of research investigating the role of honey in the treatment of various diseases, including cancer. These health benefits of honey in treating diverse diseases can be attributed to its various pharmacologically active constituents, especially flavonoids and phenolic constituents. Some of the flavonoids and phenolic compounds that have been identified in honey include chrysin, kaempferol, quercetin, pinobanksin, pinocembrin, luteolin, apigenin, genistein, naringenin, hesperetin, *p*-coumaric acid, gallic acid, ellagic acid, ferulic acid, syringic acid, caffeic acid and vanillic acid [15,21,22,23]. These honey constituents have been shown to exert anti-inflammatory, antioxidant, antiproliferative, antitumor, antimetastatic and anticancer effects [23,24,25,26]. The inhibitory effect of honey on tumorigenesis and cancerogenesis can therefore be attributed to the presence of these flavonoids and phenolic acids. This review article highlights the role of honey in modulating the development and progression of tumor or cancer as well as various possible mechanisms by which honey may inhibit growth of cancer. Findings on the antimetastatic, antiproliferative and anticancer effects of honey in various forms of cancer such as breast and colorectal cancer are discussed. In contrast, limited but promising data are available for other forms of cancers including prostate, bladder, endometrial, skin, cervical, oral and bone cancer cells. While honey is selectively toxic to tumor or cancer cells, it is non-cytotoxic to normal cells. Hence, honey may be used as a cancer therapeutic agent or to complement conventional cancer treatments.

## 2. Structures of Major Flavonoids and Phenolic Acids in Honey

**Figure 1:**
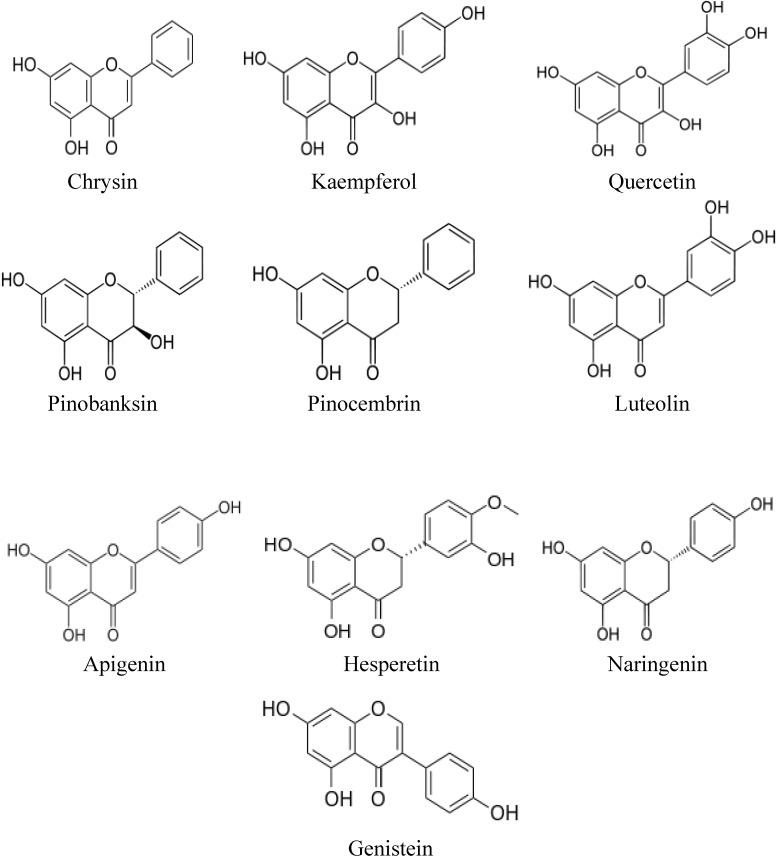
Chemical structures of flavonoids in honey.

**Figure 2:**
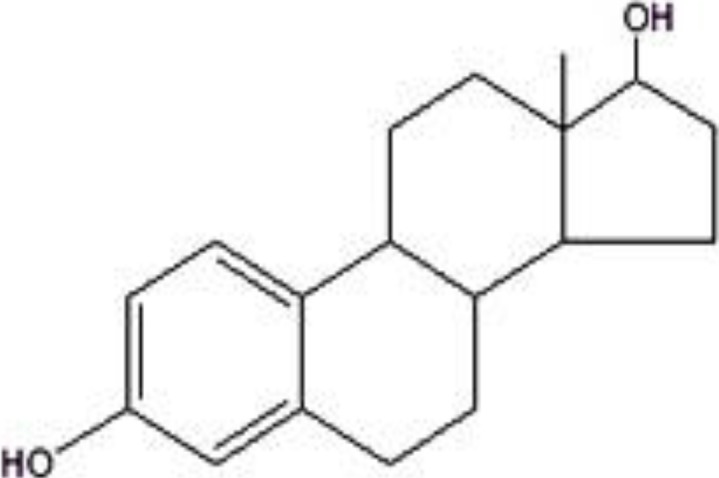
Chemical structure of the endogenous estrogen 17-β-estradiol.

**Figure 3:**
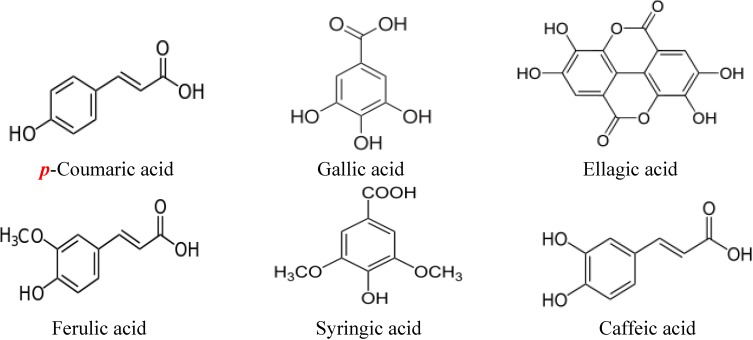
Chemical structures of phenolic acids in honey.

Flavonoids refer to a group of biologically active natural products with a 15-carbon (C6-C3-C6) structure, comprising two benzene rings joined by a heterocyclic pyrane ring [27]. They are generally classified as flavonols (e.g., kaempferol, quercetin and pinobanksin), flavones (e.g., chrysin, luteolin and apigenin), flavanones (e.g., pinocembrin, naringenin and hesperetin), isoflavones (e.g., genistein) and anthocyanidins [28]. Some flavonoids such as chrysin, genistein, naringenin and luteolin have been shown to exhibit estrogenic activity and are often referred to as phytoestrogens [29]. The chemical structures of some of the major flavonoids present in many varieties of honey are shown in Figure 1. Figure 2 shows the chemical structure of 17-β-estradiol (an endogenous estrogen) revealing its similarity to that of the flavonoids. The chemical structures of some of the phenolic acids in honey are shown in Figure 3.

## 3. Effects of Honey on the Development and Progression of Tumor and Cancer

There has been an increase in the number of studies investigating the potential role of honey in the prevention of tumor or cancer development and progression. Most of these researches utilize *in vitro* techniques, while a scarcity of studies employ *in vivo* models. These effects of honey on the development or progression of tumors or cancers are highlighted according to the type of tumor or cancer in the following subsections.

### 3.1. Breast Cancer

Breast cancer is the major cause of cancer deaths among women globally. It is estimated that about 12% of women will develop breast cancer in their lifetime [1]. Besides several other factors, the circulating levels of estrogens and dysregulated estrogen signaling pathways play a predominant role in the development and progression of breast cancer [30]. As a result, breast cancer therapy often targets the estrogen receptor (ER)-signaling pathway. There have been some attempts to investigate if honey could modulate this important pathway. Tsiapara and colleagues evaluated the potential of Greek thyme, pine and fir honey extracts to modulate the estrogenic activity and cell viability of breast cancer cells (MCF-7) [31]. The authors found that the honey samples exhibited a biphasic activity in MCF-7 cells depending on the concentration—an antiestrogenic effect at low concentrations and an estrogenic effect at high concentrations. In the presence of estradiol, thyme and pine honey extracts were found to antagonize estrogen activity, while fir honey extract enhanced estrogen activity in MCF-7 cells. The study also reported variations on the effects of the three honey extracts on cell viability. While the study found no effect of thyme and pine honey on MCF-7 cells, fir honey enhanced the viability of MCF-7 cells. These dual effects of honey extracts are mostly likely due to their high contents of phenolic compounds such as kaempferol and quercetin. Phenolic compounds are phytoestrogens which exert dual actions—both inhibitory and stimulatory effects [28]. Phytoestrogens are phytochemicals which are structurally similar to mammalian estrogens and therefore can bind to estrogen receptors [32]. They can elicit estrogenic or antiestrogenic effect depending on certain factors such as its concentration [32,33]. Quercetin has been reported to elicit apoptotic effects through ER α- and ER β-dependent mechanisms [34,35]. It is unclear why only fir honey but not thyme and pine honey enhanced the viability of MCF-7 cells. Further studies may reveal differences in the composition of these honey samples. It is possible that fir honey contain greater amounts of nutrients such as phenolic compounds, amino acids, vitamins, minerals and enzymes especially glucose oxidase which generate moderate levels of ROS. All this may enhance the viability of MCF-7 cells.

The cytotoxic effect of tualang honey has also been demonstrated in the human breast cancer cell lines MCF-7 and MDA-MB-231 [36]. The cytotoxicity was evident by increased leakage of lactate dehydrogenase (LDH) from the cell membranes. Tualang honey was shown to induce apoptosis and reduce mitochondrial membrane potential. The authors also found that honey exerted no cytotoxic effect in MCF-10A, a normal breast cell line. This therefore suggests that the cytotoxic effect of tualang honey is specific and selective to the breast cancer cell lines. This is important because selectivity and specificity are key characteristics of a good chemotherapeutic agent. Unfortunately, most anticancer drugs lack these properties. These findings have been recently confirmed in another study which compared the effect of tualang honey with that of tamoxifen (an estrogen receptor antagonist) in MCF-7 and MDA-MB-231. In addition to corroborating the previous findings [36], the study found that the anti-cancer effect of tualang honey on breast cancer cells was similar to that of tamoxifen [37]. Cytotoxic effects on breast cancer cells (MCF-7) have also been reported for Indian honey [38]. These studies reveal that honey is able to exert cytotoxicity in both MCF-7 and MDA-MB-231 which are ER-positive and ER-negative breast cancer cells, respectively. This can be attributed to the flavonoids and phenolic compounds in honey. These constituents which are phytoestrogens have been shown to stimulate both ER-α and –β subtypes [39].

Several studies have also confirmed the antimetastic, antiproliferative and anticancer effects of honey on breast tumor or cancer in rodents. In a murine (mammary carcinoma) tumor model, the anti-metastatic effect of honey when applied before tumor-cell inoculation has been reported [40]. The antimetastatic effect of honey may be due to its flavonoids such chrysin which have been shown to inhibit the metastatic potential of human breast cancer cells [41]. Similarly, a study investigated the antitumor effect of two honey samples containing different phenolic contents against Ehrlich ascites and solid carcinoma. Both honeys were found to markedly inhibit the growth of Ehrlich ascites carcinoma, but the honey containing higher phenolic content exerted a greater antitumor effect [42]. Research carried out by Tomasin and Gomes-Marcondes investigated the effects of combined *Aloe vera* and honey on tumor growth and cell proliferation against Walker 256 carcinoma implant in Wistar rats. Both agents were found to suppress tumor growth and inhibit cell proliferation [43].

In a recent study, Abd Kadir and colleagues investigated the inhibitory effect of Malaysian tualang honey on the development of 7,12-dimethylbenz(α)anthracene (DMBA)-induced breast cancer in rats. The researchers found that the untreated DMBA-induced breast cancer rats (control rats) showed tumor development much earlier than the honey-treated DMBA-induced breast cancer rats. The control rats also showed marked increase of tumor size over a shorter period of time. In contrast, the size increment and tumor size were markedly reduced in honey-treated DMBA-induced breast cancer rats compared to the untreated cancer rats. The authors also found that the honey-treated cancer rats had fewer numbers of tumors than the controls. Though not statistically significant, increasing the dose of honey tended towards increased apoptotic index. While most of the honey-treated cancer rats developed low or medium grade tumor, the untreated rats had mostly high grade tumors. The histological analysis also revealed that the cancer cells from the honey-treated rats were more identical, with denser nuclei, while those of the control rats had more pleomorphic cells with more prominent nuclei. Other findings include less prominent vasculature around the tumor nodules and significantly reduced weights and volumes of the tumor masses (which were smaller, softer and paler with spots of necrosis) in honey-treated rats [44].

These data suggest that honey exhibits anticancer effect as evident by its antiestrogen activity and potential in inducing mitochondrial membrane depolarization and apoptosis in breast cancer cells. The antiproliferative and antimetastatic effects of honey are also demonstrated by its ability to suppress tumorigenesis and reduce size and number of tumors in DMBA-induced breast cancer in rats. The data also support previous observations that the biological or pharmacological effects of honey may vary among honey samples and dose-dependent. Additional data, especially emanating from *in vivo* studies, are necessary to support or strengthen the anticancer effect of honey in breast cancer.

### 3.2. Liver Cancer

Like other cancers, the prevalence of liver cancer is on the rise. A 2005 report estimated that 82% of liver cancer cases occurred in developing countries [45]. Hepatocellular carcinoma (HCC) is the most predominant liver cancer. The increased incidence of HCC is linked to various factors, mainly infection with hepatitis B or hepatitis C virus, as well as diabetes, obesity, hereditary and social risk factors such as excessive consumption of alcohol [46]. In spite of considerable advances in the treatment of HCC, the quality of life among patients remains very poor. This suggests the need to continuously strive for better therapies. The antitumor effects of honey on liver cancer cells have recently been reported in a number of studies. Hassan *et al.* demonstrated that treatment of human hepatocellular carcinoma (HepG2) cells with honey markedly reduced the number of viable HepG2 cells and nitric oxide (NO) levels, while it enhanced the total antioxidant status (TAS) [47]. Based on these findings, it can be speculated that the viability or survival of HepG2 cells is sustained by reactive oxygen species (ROS). Moderate levels of ROS enhance cell proliferation, growth and differentiation [48,49]. The reduced levels of NO following honey treatment lend credence to this view. By scavenging ROS, honey will invariably enhance TAS as shown in this study. Hence, decreased ROS and improved antioxidant defenses will consequently lead to inhibition of proliferation as evidenced by the reduced number of viable HepG2 cells. A study that investigated the antiproliferative effects of gelam honey on HepG2 found that the IC_50_ value of gelam honey towards HepG2 was 25% whereas it was 70% for normal human hepatocytes (WRL-68) [50]. This shows gelam honey is selectively cytototoxic to liver cancer cells. The study also revealed that gelam honey inhibited the proliferation of HepG2 cells and induced apoptosis in HepG2. A similar study evaluated the antiproliferative effect of a mixture of gelam honey and *Tinospora crispa* on HepG2 and WRL-68 cells [51]. The study reported an IC_50_ value of 42.67% in HepG2 cells and none in the WRL-68 cells. The mixture was also found to induce apoptosis in HepG2 cells. Antiproliferative effect of Thailand pot-honey on HepG2 cells has also been demonstrated [52].

A study by Abdel Aziz and colleagues investigated the effects of honey extracts on HepG2 cell lines [53]. The authors reported that honey extracts exerted cytotoxic, antimetastatic and anti-angiogenic effects in HepG2 cells. These effects were found to vary with different degrees based on the honey quality while some honey samples with poor quality did not produce cytotoxic effect. Some of the honey extracts were also found to enhance proliferation of cancer cells—an effect that may be due to the nutrients such as amino acids, minerals, vitamins and antioxidants in honey. Availability of such nutrients may serve as substrate for cellular metabolism in HepG2 cells. The proliferative effect of honey may also be due to hydrogen peroxide generated by honey. Peroxide-producing honey has been reported to liberate radicals [54]. Several factors may influence the amount of ROS generated by honey. For instance, honey samples that contain higher amounts of the enzyme glucose oxidase may produce more ROS. Similarly, the duration and techniques of storage may also influence the level of ROS formed. Though the concentrations may be small, such small amounts of ROS may stimulate cancer cell proliferation. While this potential proliferative effect may likely be suppressed in honey rich in phenolic compounds, it may be augmented in honey with low phenolic compounds. This may explain the variations of the anticancer properties of the different honey extracts. Findings from Hanaa and Shaymaa’s study also confirmed the antitumor effect of honey (which was enhanced by adiponectin) in HepG2 cells [55]. A recent study investigated the protective effect of three honey samples of diverse floral origin (rosemary, heather and heterofloral) on mutagens-induced DNA strand breaks in a HepG2 cell line [56]. The study utilized N-nitrosopyrrolidine (NPYR), N-nitrosodimethylamine (NDMA), benzo(a)pyrene (BaP) and 2-amino-1-methyl-6-phenyl-imidazo[4,5-b]pyridine (PhIP) as dietary mutagens. The results showed that honey samples protected against DNA damage induced by NPYR, BaP and PhIP but not NDMA, whereas artificial honey exerted no such protective effect. The study found an association between high phenolic content and protection of HepG2 against mutagens-induced DNA damage. The protective effect of honey against mutagens-induced DNA damage in HepG2 can be attributed to its antioxidant and free radical scavenging properties.

There are limited data on the potential inhibitory effects of honey on hepatic tumorigenesis or carcinogenesis *in vivo*. A recent study investigated the effect of honey on the development and progression of diethylnitrosamine (DEN)-induced hepatic cancer in rats [57]. After treatment for six months, the liver of untreated DEN-injected rats showed a variety of lesions including inflammatory lymphocytic infiltration, fatty degeneration with displacement of the nucleus, oedema and injured hepatocytes with hyperchromatic nuclei. The liver of DEN-injected rats also showed the presence of neoplastic hepatic cells which were polyhedral to round with dense vesicular nuclei. Several strong positive stained nuclei for p53 and PCNA expressions were also observed in the liver of untreated DEN-injected rats. These abnormalities including neoplastic hepatic cells, stained nuclei for p53 and PCNA expressions were considerably reduced in the liver of honey-treated DEN-induced rats. These findings suggest that honey has an anticancer effect on liver cancer cells and exerts a protective effect against chemical-induced hepatocarcinogenesis in rats.

### 3.3. Colorectal Cancer

Colorectal cancer accounts for about 9% of all cancer cases [58]. It is the third commonest cancer in the World and the fourth most frequent cause of death [59]. Environmental factors have been identified to play a predominant role in the pathophysiology of colorectal cancer [60]. In spite of advances in its treatment, including postoperative care, recurrence and mortality rates remain high [61], hence the urgent need to complement the current therapies. A study that investigated the chemopreventive effects of gelam and nenas monofloral honeys against colon cancer cell lines (HT 29) found that the honey samples inhibited proliferation of colon cancer cells. Both honeys also caused DNA damage in a dose dependent manner and suppressed inflammation in H_2_O_2_ inflammation-induced colon cancer cells [62]. In a recent study, Jaganathan and Mandal investigated the apoptotic effect of some crude honey samples in colon cancer cell lines, HCT 15 and HT 29. The study confirmed the antiproliferative effect of honey in colon cancer cells as previously reported and also revealed that this effect was dependent on the level of phenolic content. The higher the phenolic content, the greater the antiproliferative effect against colon cancer cells [63]. In addition to its antiproliferative effect, honey also induced apoptosis by causing the depletion of intracellular non-protein thiols. It also reduced the mitochondrial membrane potential and increased generation of ROS [64]. Findings from *in vivo* studies have also demonstrated the anticancer effect of honey. Studies by Orsolić *et al.* showed that honey, when applied before tumor-cell inoculation, exerted anti-metastatic effect in a murine tumor model with colon carcinoma [40,65]. This suggests that the mice are better protected against tumor development when honey is administered before tumor-cell inoculation. In other words, the anti-metastatic effect of honey in this murine tumor model is prophylactic. These findings deserve to be investigated further especially using other tumor models. Similarly, the antimetastatic effect of honey has been demonstrated in anaplastic colon adenocarcinoma of Y59 rats [66]. Other studies have also confirmed the protective effect of honey against methylnitrosourea (MNU)-induced colon adenocarcinoma in rats [67].

### 3.4. Prostate Cancer

A study that investigated the bioactivity of three Greek honeys (thyme, pine and fir honey) on prostate cancer cells (PC-3) found marked differences in the effects of honeys on cell viability [31]. The study showed that only thyme honey reduced considerably the viability of PC-3 cells while no such effect was found for pine and fir honey extracts. Iranian honey has also been shown to induce apoptosis and inhibit proliferation of PC-3 cells [26]. These findings suggest that honey exerts antiproliferative effect on prostate cancer cells. The data also reveal that not all honey samples exhibit antiproliferative effect. This seems to support previous findings that the effect of honey on cell proliferation is dependent on the concentration of honey as well as the cancer cell line.

### 3.5. Other Forms of Cancer

Swellam and colleague reported that honey markedly inhibited the proliferation of three human bladder cancer cell lines—T24, 253J and RT4, as well as one murine bladder cancer cell line, MBT-2 [68]. The authors also investigated the *in vivo* effect of honey on bladder cancer cells implanted subcutaneously in the abdomens of mice. It was shown that administration of 6% and 12% honey via intralesional and oral routes significantly inhibited tumor growth. Honey has also been reported to inhibit cell proliferation, induce apoptosis, alter cell cycle progression and cause mitochondrial membrane depolarization in many other forms of cancer including endometrial cancer cells [31], renal cell carcinoma [69], skin cancer cells (melanoma) [70], cervical cancer cell lines [37], human non-small cell lung cancer cells [71], mouth cancer cells (oral squamous cell carcinoma) and bone cancer cells (osteosarcoma) [72]. In a recently published article, Morales and Haza investigated the antiproliferative and apoptotic effects of three crude Spanish honey samples of different floral origin (polyfloral, heather and rosemary honey) and those of an artificial honey in HL-60, a human peripheral blood promyelocytic leukemia cell line [73]. The researchers reported that all the three honey samples induced apoptosis, in a concentration and time dependent-manner. The apoptotic effect of the honey samples was also found to be dependent on the levels of their phenolic content. The antimetastic effect of honey has also been demonstrated in mice with transplantable tumors [65]. A study reported a positive effect of honey ingestion on gastric cancer via induction of apoptosis in gastric mucosa [74]. The summary of the effects of honey on the development and progression of tumor and cancer is presented in Table 1. The table shows the type of cancer or tumor in which the effects of honey have been investigated. It also reveals the key findings on the effects of honey on these tumor or cancer cells.

**Table 1:** Effects of honey on the development and progression of tumor and cancer cells.

Type of tumor/cancer and cancer cell type	Effects of honey (key findings)	References
*In vitro* **studies**		
Human breast cancer (MCF-7 & MDA-MB-231)	Antagonizes estrogen activity, inhibits cell proliferation, induces apoptosis, reduces mitochondrial membrane potential	[31,36,37,38]
Human liver cancer (HepG2)	Inhibits cell proliferation, suppresses angiogenesis, induces apoptosis, protects against mutagen-induced DNA damage	[47,50,52,53,55,56]
Human colorectal cancer (HT 29, HCT 15 & CT 26)	Inhibits cell proliferation, induces apoptosis, arrests cell cycle, reduces mitochondrial membrane potential, increases generation of ROS, depletes intracellular non-protein thiols, induces DNA damage, suppresses inflammation	[62,63,64,75]
Human prostate cancer (PC-3)	Inhibits cell proliferation, induces apoptosis	[26,31]
Human bladder cancer (T24, 253J, RT4 & MBT-2)	Inhibits cell proliferation	[68]
Human kidney cancer (Renal cancer cell line)	Induces apoptosis	[69]
Human oral cancer (Oral carcinoma)	Inhibits cell proliferation	[72]
Human bone cancer (Osteosarcoma)	Inhibits cell proliferation	[72]
Human skin cancer (Melanoma cells)	Inhibits cell proliferation, arrests cell cycle	[70]
Human leukemia	Induces apoptosis	[73]
Human endometrial cancer	Inhibits cell proliferation	[31]
Human lung cancer NCI-H460	Inhibits cell proliferation	[71]
Human cervical cancer	Induces apoptosis, disrupts mitochondrial membrane potential	[36]
*In vivo* **studies**		
Walker 256 carcinoma	Inhibits cell proliferation, arrests cell cycle, induces apoptosis	[43]
DMBA-induced breast cancer in rats	Delays the development of tumors, reduces the number and size of tumors, prevents the development of high grade cancer	[44]
Rats with DEN-induced hepatic cancer	Protects against transformation of normal liver cells to neoplastic hepatic cells, restores the PCNA and P53 expression	[57]
Mice/rats with colon carcinoma or adenocarcinoma	Inhibits formation of metastases and tumor growth	[40,65,66,67]
Mice implanted with bladder cancer cells	Inhibits tumor growth	[68]
Mice with melanoma	Inhibits tumor growth, induces apoptosis	[75]

## 4. Mechanisms of the Antiproliferative, Antimetastatic and Anticancer Effects of Honey

Some of the mechanisms by which honey may exert its antiproliferative, antimetastatic and anticancer effects are discussed in this section. These include, but are not limited to cell cycle arrest, activation of mitochondrial pathway, induction of mitochondrial outer membrane permeabilization, induction of apoptosis, modulation of oxidative stress, amelioration of inflammation, modulation of insulin signaling, and inhibition of angiogenesis.

### 4.1. Cell Cycle Arrest

Cell cycle is a series of coordinated events in which cell growth and proliferation are tightly controlled. It comprises four sequential phases—G_1_, S, G_2_ and M. DNA replication takes place at the S phase while the cell divides into two identical daughter cells at the M phase [76]. The G_1_ and G_2_ are the gap phases between S and M. At the G_1_ phase, the cells are responsive to extracellular signals by progressing towards mitosis or withdrawal from the cell cycle into a quiescent stage known as G_0_[77]. The regulation of the cell cycle events is under the control of a cascade of protein kinases and checkpoints [78]. In cancer cells, the cell cycle becomes dysregulated and this results in uncontrolled cell proliferation. Using various cancer cell lines, honey has been documented to induce cell cycle arrest. Honey treatment of bladder cancer cell lines was shown to cause a considerable arrest of cell cycle in the sub-G_1_ phase [68]. Recently, Aliyu and colleagues also showed that the cytotoxic effect of honey against non-small cell lung cancer cell (NCI-H460) was mediated via arrest of cell cycle at G_0_/G_1_ phase [79]. Available data reveal that the ability of honey to arrest cell cycle is due to the various flavonoids and phenolic compounds in honey. In one of such studies, Pichichero *et al.* [70] found both honey and its constituent chrysin exerted antiproliferative effects in human and murine melanoma cells via cell cycle arrest at G_0_/G_1_ phase. Besides chrysin, several other phenolics such as quercetin and kaempferol which are found in large quantities in honey have also been shown to arrest cell cycle at various phases such as G0/G1, G1 and G2/M in human melanoma, renal, cervical, hepatoma, colon and oesophageal adenocarcinoma cell lines [80,81,82,83].

### 4.2. Activation of the Mitochondrial Pathway

One of the mechanisms by which chemotherapy and radiotherapy cause cancer cell death is the activation of the mitochondrial pathway [84]. Mitochondrial membrane permeabilization is an early event in the mitochondrial pathway, also known as intrinsic pathway. The mitochondrial pathway involves a series of interactions between several stimuli including nutrients, physical stresses, oxidative stress and damage [85], during which several proteins (such as cytochrome c) usually located in the intermembrane mitochondria space (IMS) become released resulting in cell death [86]. Therefore, compounds or agents such as honey rich in flavonoids that are capable of activating mitochondrial pathway and release of proteins such as cytochrome C are considered potential cytotoxic agents [87,88].

### 4.3. Induction of Mitochondrial Outer Membrane Permeabilization

The induction of mitochondrial outer membrane permeabilization (MOMP) leads to leakage of intermembrane space proteins into the cytosol and consequently causing cell death [89]. Induction of MOMP is a common mechanism of agents with anticancer properties. Honey induces mitochondrial membrane permeabilization in various cancer cell lines via reduction of the mitochondrial membrane potential [36]. Honey has also been found to augment the apoptotic effect of tamoxifen via increased depolarization of the mitochondrial membrane [37]. Similarly, a study by Jaganathan and Mandal found that reduction of mitochondrial membrane potential is an important mechanism of the cytotoxicity of Indian honey in HCT-15 and HT-29 colon cancer cells [64]. Flavonoids such as quercetin have been shown to cause MOMP [90]. Therefore, it can be inferred that cancer cell death induced via MOMP by honey is mediated partly through its flavonoid constituents.

### 4.4. Induction of Apoptosis

Apoptosis is a programmed cell death which helps to regulate cell growth and eliminate damaged cells [91]. Several apoptotic pathways are deregulated in cancer cells and this favors their survival and immortality [92]. The apoptotic pathway involves MOMP which leads to the release of IMS pro-apoptotic proteins such as cytochrome c which in turn activate caspase cascade resulting in mitochondrial dysfunction and cell death [93,94]. Treatment of cancer cells with honey was shown to cause apoptotic cell death in breast cancer cells via induction of caspase-3/7 and -9 activation [36]. Honey was also recently reported to enhance tamoxifen-induced apoptosis by activating caspase-3/7, -8 and -9 [37]. The effect of honey has also been demonstrated on several enzymes, genes and transcription factors related to apoptosis. Colorectal cancer cell lines HCT-15 and HT-29 treated with honey showed down-regulation of poly(ADP-ribose) polymerase (PARP) expression [64]. The PARP is an enzyme that plays a vital role in apoptosis and DNA repair [95]. The inhibition of PARP activity by honey will prevent DNA repair and thereby contribute to increased cytotoxicity of honey in cancer cells. The study further revealed that honey treatment induced or activated caspase-3, p53 and Bax expression while it down-regulated Bcl2 expression. Honey has also been shown to exert anti-mutagenic effect by inhibiting error-prone repair pathway [96].

The p53, also known as tumor suppressor p53, is an important transcription factor regularly inactivated in many forms of human tumors [97,98]. It mediates tumor suppression by modulating the transcription of many genes involved in the regulation of apoptosis [99]. Honey treatment in diethylnitrosamine (DEN)-induced carcinogenic rats was reported to be associated with nearly normal hepatocytes and few neoplastic cells whereas the untreated carcinogenic rats were characterized by severely injured hepatocytes, several neoplastic cells and up-regulated p53 expression [57]. The study showed that the antineoplastic effect of honey was mediated via restoration of p53 expression. Tomasin *et al.* who evaluated the effect of honey and *Aloe vera* on apoptosis in rats with Walker 256 carcinoma reported that tumors from rats treated with honey and *Aloe vera* had markedly higher Bax/Bcl-2 ratio [43]. Similarly, Fernandez-Cabezudo and colleagues demonstrated the apoptotic effect of honey on human breast cancer, murine melanoma and colorectal carcinoma cells. The apoptotic effect of honey was found to be mediated via the activation of caspase 9, caspase 3 and down-regulated Bcl-2 expression [75]. The apoptotic effect of honey is most likely due to its phenolics. Quercetin has been reported to inhibit pancreatic and breast cancer cell growth and induce apoptosis via Bcl-2 expression downregulation and upregulation of Bax expression [100,101]. Recent evidence reveals that chrysin, a key constituent of honey, exerts antimetastatic effect in human breast cancer cells [41]. Similarly, chrysin was shown to induce apoptosis through caspase-3 and Bax activation in B16-F1 and A375 melanoma cells [102]. The Bcl-2 is an anti-apoptotic protein commonly over-expressed in many forms of cancers [103]. On the other hand, Bax is a pro-apoptotic protein. Cancer cells generally have characteristic deregulated or impaired apoptosis resulting from down-regulation of pro-apoptotic proteins and/or up-regulation of anti-apoptotic proteins [104]. Hence, these findings reveal that honey induces cancer cell death or apoptosis via activation of caspase cascade, induction of p53 and up-regulation of pro-apoptotic proteins—Bax and down-regulation of anti-apoptotic proteins such as Bcl-2. The data also point to the role of honey flavonoids in the apoptotic effect of honey in cancer cells.

### 4.5. Modulation of Oxidative Stress

The role of reactive oxygen species (ROS) and oxidative stress in cancer growth and inhibition still remains controversial. There is evidence in support of dual roles of ROS (stimulatory and inhibitory roles) in cancer. Low levels of ROS enhance proliferation of cells [105]. On the other hand, increased levels of ROS which cause oxidative damage are well documented in many forms of cancer such as colorectal cancer [106], breast cancer [107], lung cancer [108,109] and gastric cancer [110]. Therefore, the maintenance of redox homeostasis is important for normal cell growth and proliferation. Considering that ROS are double-edged sword, available evidence also suggests that selective exposure of cancer cells to increased levels of ROS and/or lipid peroxidation products may result in cancer cell death [111,112]. Honey is a potent antioxidant and free radical scavengers [113,114]. Several of its biological effects are attributed to its antioxidant properties [14,115,116]. Therefore, the inhibitory effect of honey on cancer growth and proliferation may be mediated partly via its modulation of oxidative stress.

Data from Hassan and colleagues’ study indicated that the anti-neoplastic effects of honey and *Nigella sativa* on hepatocellular carcinoma cells were associated with improved antioxidant status [47]. On the other hand, Jaganathan and Mandal showed that anticancer effect of honey is mediated via increased oxidative stress. The researchers reported that honey treatment caused non-protein thiol depletion in HCT-15 and HT-29 colon cancer cells. The antiproliferative effect of honey was also shown to be associated with increased generation of ROS. Honey-induced cell death was accompanied by DNA fragmentation, which was markedly inhibited by treatment with antioxidant, N-acetyl-L-cysteine (NAC) [64]. By and large, these findings indicate honey can inhibit cancer growth by modulating oxidative stress—that is, via amelioration or induction of oxidative stress. Whether honey exerts anticancer effect via antioxidant or pro-oxidant mechanism seems to largely depend on the oxidative stress status in the cancer cells. If survival of cancer cells is dependent on low level of ROS and oxidative stress, honey may act as a pro-oxidant to generate more ROS and increase oxidative stress. On the other hand, if cancer growth is sustained or enhanced by elevated levels of ROS and oxidative stress, honey acts as an antioxidant by scavenging ROS and reducing oxidative stress. In both cases, pro-oxidant and antioxidant effects of honey invariably result in cancer cell death. These dual effects of honey in cancer cells are mostly likely due to its phenolic constituents. Besides their antioxidant properties, phenolic compounds are easily oxidizeable. Beverages which have high concentrations of polyphenols and strong antioxidant properties have been reported to generate high H_2_O_2_ levels upon exposure to air [117]. Besides, following their interactions with transition metal ions such as copper, honey phenolic compounds may liberate ROS causing ROS-mediated DNA damage and resulting in cell death [118,119]. Several flavonoids and phenolics have been demonstrated to induce ROS in cancer cells [120]. However, a recent study revealed that while the concentrations of phenolic compounds play a key role in honey-induced HL-60 cell death, honey did not generate ROS and NAC (a potent antioxidant) did not prevent honey-induced cancer cell death [73]. This suggests that in spite of its high phenolic content, honey can still exert its anticancer effect via ROS-independent mechanism(s).

### 4.6. Amelioration of Inflammation

Chronic inflammatory disorders are associated with increased risk of cancer. The development of inflammatory-related/induced cancers usually occurs within the vicinity of the affected cells or tissues. Colorectal cancer, for example, may develop from ulcerative colitis or inflammatory bowel diseases [121]. Infection caused by schistosomes and other parasites may predispose the individual to bladder cancers [122]. Likewise oral infections such as caused by human papillomavirus (HPV) may lead to oropharyngeal cancer [123]. Inflammation is also an important factor in the pathophysiology of many other cancers/malignancies [124,125]. Two important components of the inflammatory pathway frequently activated in cancers are mitogen-activated protein kinase (MAPK) and nuclear factor kappa B (NF-kB) pathways [126]. Activation of MAPK and/or NF-κB, subsequently results in induction of several inflammatory proteins and genes including cyclooxygenase-2 (COX-2), C-reactive protein (CRP), lipoxygenase-2 (LOX-2), and pro-inflammatory mediators or cytokines such as interleukin 1 (IL-1), IL-6 and TNF-α. All these pathways and pro-inflammatory mediators are known to play an important role in angiogenesis and inflammatory related etiology of cancer [127,128]. Diverse biological processes and events that enhance inflammation can contribute to tumorigenesis because chronic inflammation is a key driver of promotion, the second stage of cancer development. On the other hand, biological processes and activities that decrease inflammation will not only hinder or limit the promotion stage (tumorigenesis) but also prevent the transition of this stage to progression stage (carcinogenesis). In other words, amelioration of inflammation can help to prevent both the formation of a benign tumor and its progression to malignant cancer. Hence, several researchers are paying more attention to the prospect of treating cancer by targeting inflammation [129].

Batumalaie *et al.* recently showed the honey treatment reduced the expression of MAPK and NF-κB in HIT-T15 cells [130]. Chrysin, commonly found in honey, has also been shown to induce apoptosis via modulation of MAPK in B16-F1 and A375 melanoma cells [102]. A study investigated the effect of honey flavonoid extract (HFE) on the production of pro-inflammatory mediators by lipopolysaccharide-stimulated N13 microglia. The data indicated that HFE markedly suppressed the release of pro-inflammatory cytokines including TNF-α and IL-1β [23]. Chrysin was also shown to enhance TNF-related apoptosis-inducing ligand (TRAIL) induced apoptosis in cancer cell lines [131]. Honey also suppressed edema and leukocyte (monocyte and neutrophil) infiltration [132]. Recently, evidence has also emerged from *in vivo* studies demonstrating the anti-inflammatory effect of honey. Hussein *et al.* evaluated the anti-inflammatory effect of honey in rats with carrageenan-induced inflammation [133]. The study showed that honey decreased edema in a dose-dependent manner while it reduced the plasma levels of inflammatory mediators including IL-6, TNF-α, PGE2 and NO. The authors also reported that honey administration inhibited the expression of IL-6, TNF-α, iNOS and COX-2 in paw tissue. The anti-inflammatory of honey was found to be comparable to that of indomethacin. In their latest findings, the researchers demonstrated that honey markedly attenuated NF-κB translocation to the nucleus and suppressed IκBα degradation [134]. The cytotoxic effect of honey against non-small cell lung cancer cell was also reported to be partly mediated via modulation of inflammatory cytokines [79]. These findings suggest the anti-inflammatory effect of honey, mediated via attenuation of pro-inflammatory mediators and inhibition of NF-κB and MAPK signaling pathways, may contribute considerably to the anticancer effect of honey. The anti-inflammatory effect of honey can be attributed to its phenolic compounds and flavonoids [15,135,136].

### 4.7. Modulation of Insulin Signaling

Evidence from epidemiological studies indicates insulin resistance, type 2 diabetes mellitus and obesity are major risk factors for different types of tumors or malignancies [137,138,139]. Research in the past few years has implicated the role of insulin receptor (IR) in cancerogenesis [140]. Vincent *et al.* recently demonstrated that small molecule inhibitors that target both the IR and insulin-like growth factor-1 receptor (IGF1R) are effective in reducing non-small cell lung cancer cell proliferation [141]. Another key component of insulin signaling is the PI3K/Akt. The PI3K/Akt is known for its role in modulating the functions of several substrates that regulate cell survival, cell cycle progression and cellular growth [142]. The effect of gelam honey extracts on Akt activated insulin signaling pathway in HIT-T15 cells under hyperglycemic conditions was recently investigated [130]. The researchers reported that the development of insulin resistance was characterized by increased levels of MAPK, NF-κB, and insulin receptor substrate 1 (IRS-1) serine phosphorylation while Akt expression and insulin contents were markedly reduced. The study showed that pretreatment with gelam honey and quercetin extracts improved insulin resistance and insulin content. Honey treatment increased the expression of Akt while it reduced the expression of IRS-1 serine phosphorylation, MAPK and NF-κB. These findings suggest that honey can modulate insulin signaling. Such an effect may contribute to its anticancer effect. Hence, similar studies are necessary in cancer cells.

### 4.8. Inhibition of Angiogenesis

Cancer cells generally are capable of penetrating blood or lymphatic vessels. They are circulated and later proliferate at adjacent or distant sites/organs, a process known as metastasis [143]. Metastasis requires the growth of the vascular network which is necessary for the provision or delivery of oxygen, cellular metabolic substrates including nutrients and even blood containing the immune cells [144]. This process of formation of new blood is known as angiogenesis. Angiogenesis is a key event in the development, growth, progression and metastasis of tumor/cancer [145]. Recent research has focused on several angiogenic factors and their inhibitors in the treatment of cancer [146]. Many studies have shown that honey has a debriding effect, promotes epithelization, enhances the growth of granulation tissue and promotes angiogenic activity in the vasculature [147,148,149]. In non-cancer cells, honey may stimulate angiogenesis via generation of hydrogen peroxide [150]. The angiogenic effect of honey is perhaps beneficial in wound healing. On the other hand, it is possible that honey inhibits angiogenesis in cancer cells. The potential anti-angiogenic effect of honey in cancer cells is premised on the evidence that honey inhibits cancer cell viability, metastasis and activities of gelatinase and protease. This is evident from a study by Abdel Aziz and colleagues who demonstrated that honey exerted anti-angiogenic effects in hepatocellular carcinoma HePG2 cell lines [53]. Honey is also rich in polyphenols. Polyphenols from natural products such as red wine and green tea have been shown to exert anti-angiogenic and anticancer effects [151,152,153]. Propolis is another bee product like honey. Propolis, like honey, has anticancer effects [154] and it has been shown to inhibit tumor-induced angiogenesis [155]. Caffeic acid phenethyl ester (CAPE), chrysin and other cytotoxic constituents of honey have been reported to exert anti-angiogenic and anticancer effects [25,135,156]. These data therefore indicate that honey can exert it anticancer effect via inhibition of angiogenesis.

**Figure 4:**
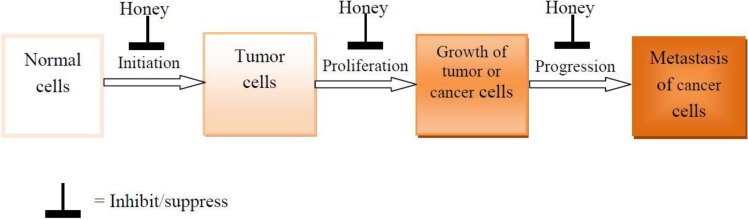
Honey blockage of the 3 stages of cancerogenesis.

**Figure 5:**
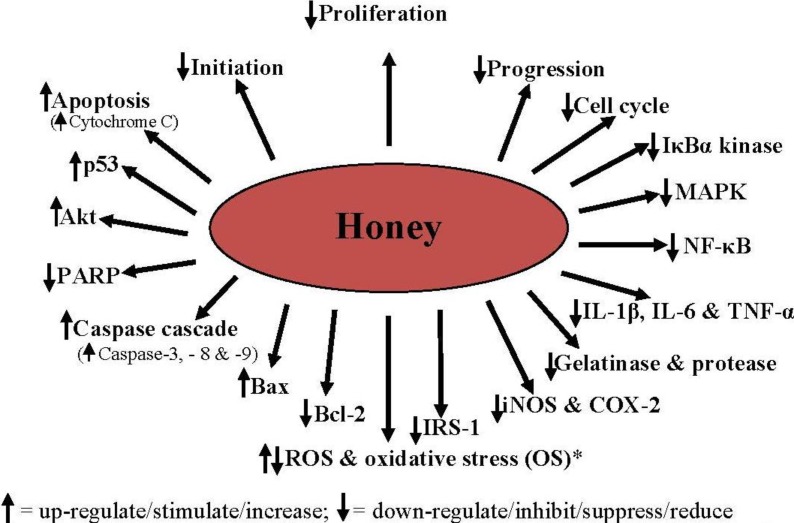
Molecular mechanisms/targets mediating the antiproliferative, antitumor, antimetastatic and anticancer effects of honey.

Honey can inhibit the development of cancer by blocking the three main stages of cancerogenesis as shown in Figure 4. The various molecular mechanisms or targets by which honey can suppress cancer development are shown in Figure 5.

Honey inhibits/suppresses the 3 stages of cancerogenesis at initiation [56,57], proliferation [43,44,63] and progression [40,65,66].

* Honey may generate ROS (and increase OS) or scavenge ROS (and reduce OS) [47,64].

## 5. Conclusions

Honey is a natural product that shows potential effects to inhibit or suppress the development and progression of tumor and cancer. Its antiproliferative, antitumor, antimetastic and anticancer effects are mediated via diverse mechanisms, including cell cycle arrest, activation of mitochondrial pathway, induction of mitochondrial outer membrane permeabilization, induction of apoptosis, modulation of oxidative stress, amelioration of inflammation, modulation of insulin signaling, and inhibition of angiogenesis in cancer cells. Honey is highly and selectively cytotoxic against tumor or cancer cells while it is non-cytotoxic to normal cells. It can inhibit cancerogenesis by modulating or interfering with the molecular processes or events of initiation, promotion, and progression stages. It, therefore, can be considered a potential and promising anticancer agent which warrants further research—both in experimental and clinical studies.
